# Supplementary material for: Patterns of Intron Gain and Loss in Fungi
Source: PLoS Biol. 2004 Nov 30;2(12):e422. doi: 10.1371/journal.pbio.0020422 (PMC532390; doi:10.1371/journal.pbio.0020422)
Supplement: Table S1 — Also available at http://genes.mit.edu/NielsenEtAl/. (4.3 MB ZIP). [file pbio.0020422.st001.zip › NielsenEtAl/html/809.html]

AN0442.1.NCU02559.1.MG03612.1.FG09305.1


```
 CLUSTAL W (1.82) Multiple Sequence Alignments - Introns Inserted


Sequence 1: NCU02559.1	209 aa
Sequence 2: MG03612.1	289 aa
Sequence 3: FG09305.1	267 aa
Sequence 4: AN0442.1	290 aa
Alignment Length: 301 aa
Number Identitical Residues: 89 aa
Alignment Score (without introns) 5461


MG03612.1 	MVKPLTFKGDK-KPKKRKRTAADDNNDDAPSSRQAATGS-------AAASEPVDEEAIAN
NCU02559.1	------------------------------------------------------------
FG09305.1 	MVKPLTFKGDK-KVKKRKRTDAEKASRDDVNDETDQQVQ-------KTTTEEAE-----N
AN0442.1  	MVKPLTFKGDKPKSKKRKNPSSTSTDFSAPAKLPKQSQSSSDLRQDQTPYSNEEAENTAE
          	  .. : ...... .. . . : . . .   .      .::.  .. :. .  . .  :.

MG03612.1 	DDSWVSAEAVNDVSGPVMFVLPTEPPSALACDAAGKVFTLPIENIVDGNPASAEPHDVRQ
NCU02559.1	-----------------MFVLPTEPPTALACDVTGKVFTLPIENIVDGNPATAEPHDVRQ
FG09305.1 	DDSWVSAEATTDVSGPIMIVLPTDTPSALACDAAGKVFTVPIENIVDGNPATAEPHDVRQ
AN0442.1  	DQSWVSADTTSDISGPILIVLPSDPLTCIASDMNGKVFASELENLIDGDARTAEPHDVRQ
          	..:  ::.: .. :.. ::***::. :.:*.*  ****:  :**::**:. :********

MG03612.1 	VFVANKIVGTENYRFKGHHGK2YLGCDKMGILSATSDAVSPLECFVLIATPDTPGTFQVQ
NCU02559.1	VWVANKIVGTENHRFKGHHGK2YLSCDKIGLFTANSDAITSLESFTIIPTFDTPGTFQIQ
FG09305.1 	VWIVNRVAGTESFRFKGHHGR2YLSCDKIGMLSADSEAVSPLESFNIIPTGDTPGTFQIQ
AN0442.1  	VWVATRVAGTEGFSLKGAHGR2YLSCDKYGLFSATASAISQHESFLIHPSPDIPGAFFMQ
          	*::..::.***.. :** **: **.*** *:::* :.*::  *.* : .: * **:* :*

MG03612.1 	TLRETFIAVKEPKARAAGASASSSKSAKAAAPEVRGDASDITFETTLRIRMQARFKPRIK
NCU02559.1	TLRDTFLTVR---------ASNSSKAN--AAPEVRGDATEISFDSTLRIRMQARFKPRIK
FG09305.1 	TLRDTFLSIK-----------NPSKAS-SKACDVRGDADTISFDTTFRIRMQARFKPKLR
AN0442.1  	TQGGTGESDT----------FVSVKEG-AKGVEIRGDENSLSFQTTLRVRMQARFKPRIK
          	*   *  :              . *   : . ::***   ::*::*:*:********:::

MG03612.1 	VSKEEKARERISRRELEEAVGRRLEDDEVRLLKRARREGDYHEKLLNLK~----IKS~KH
NCU02559.1	ASKEEKAREKISRRELEEAVGRRLEEDEVKKLKRARREGNYHEVMLDLK~----VKG~KH
FG09305.1 	ASKEEKALAKISRRELEEAAGRRLDEDEVRKLKRARREGDYHERLLEIK~----VKI1R-
AN0442.1  	ANKETKAREKISRKELEEIVGRRLNEDEVKRLRKARREGNFHEEVLDVR0DCPWRKR~IL
          	..** **  :***:**** .****::***: *::*****::** :*::: ...  *    

MG03612.1 	DKYG
NCU02559.1	DKYG
FG09305.1 	----
AN0442.1  	TGYR
          	
```
